# Rightvertical axillary incision for atrial septal defect: a propensity score matched study

**DOI:** 10.1186/s13019-022-01999-0

**Published:** 2022-10-05

**Authors:** Xiaohui Yang, Yuan Hu, Jie Dong, Peng Huang, Jinwen Luo, Guangxian Yang, Xicheng Deng

**Affiliations:** 1 Heart Center, Hunan Children’s Hospital, No. 86 Ziyuan Road, 410007 Changsha, China; 2 Department of Echocardiography and Ultrasound, Hunan Children’s Hospital, 410007 Changsha, China; 3 Pediatrics Research Institute of Hunan Province, Hunan Children’s Hospital, Changsha, China

**Keywords:** Right vertical axillary incision, Median sternotomy; congenital heart disease, Atrial septal defect, Postoperative complications

## Abstract

**Background:**

Atrial septal defect is one of the most common types of congenital heart disease. This study aims to explore the surgical and cosmetic effects of open-heart surgery with right vertical axillary incision for simple congenital heart disease in infants.

**Methods:**

From June 2018 to October 2021, children who underwent direct surgery of atrial septal defect in our department were selected for a propensity score matched study. Those with direct surgery through the right vertical axillary incision were included in the right vertical axillary incision group. According to age and weight, propensity score matching method was employed to match children from the right vertical axillary incision group with those undergoing direct surgery through median sternotomy (median sternotomy group) at a 1:2 ratio. Surgery outcomes between two groups were compared to evaluate the effectiveness and safety of right vertical axillary incision group.

**Results:**

The median incision length (median, [interquartile range]) in right vertical axillary incision group (4.8 cm, [4.0–5.0]) was shorter than that in median sternotomy group (p < 0.001). The median drainage volume of drainage tube of the right vertical axillary incision group (117.5 ml, [92.8,152.8]) was smaller than that of median sternotomy group (p = 0.021). While no residual bubbles cases in the left and right ventricles and outflow tract were present in the right vertical axillary incision group, 44% of residual air bubble rate in right ventricular outflow tract was detected in median sternotomy group (p = 0.001). Additional sedation and analgesia (p = 0.003), wound infection or poor healing (p = 0.047), thoracic deformity healing (p = 0.029) and appearance satisfaction questionnaire (p = 0.018) in the right vertical axillary incision group were better than those in the median sternotomy group.

**Conclusion:**

Right axillary vertical incision can effectively reduce surgical trauma, accelerate postoperative rehabilitation. This surgical approach also provides better cosmetic effect, which is easily accepted by children’s families and worthy of further clinical application.

## Background

Atrial septal defect (ASD) is one of the most common types of congenital heart disease (CHD) [1, 2]. This condition is one of the first open-heart operations in cardiac surgery. Early surgical treatment results are satisfactory [3] with the median sternotomy approach as the standard method to correct simple heart defects [4, 5]. However, this procedure has significant surgical trauma and suboptimal postoperative recovery outcomes. Due to poor cosmetic results and complications related to sternotomy, more surgeons are reluctant to use this method in common CHD surgery [6, 7]. With the improvement of cardiac surgery technology and the progress of anesthesia and cardiopulmonary bypass technology, novel operation methods have been developed [8–10]. In recent years, percutaneous catheter intervention, small incision closure or open-heart surgery through different approaches, open-heart surgery through full thoracoscope, robot-assisted thoracoscope technology have been implemented [11–13]. In spite of these progresses, the median thoracotomy approach under general anesthesia and hypothermic cardiopulmonary bypass is still the standard surgical treatment for atrial septal defect. The middle sternum scar is a permanent symbol for patients with heart defects, which brings inevitable psychological distress for children [8, 14–17]. In recent years, methods to reduce surgical trauma and complications during the operation and to improve cosmetic outcome have been intensely explored. The purpose of this study is to explore the surgical and cosmetic effects of open-heart surgery through right vertical axillary incision for simple CHD in infants.

## Methods

### Study design

With the approval of the Ethics Committee of Hunan Children’s Hospital, a retrospective propensity score matched study was conducted with all methods performed in accordance with the Declaration of Helsinki. Children who underwent direct surgery of atrial septal defect in our department from June 2018 to October 2021 were included. Those who underwent direct surgery through the right vertical axillary incision were defined as the right vertical axillary incision group. Children who underwent direct surgery through median sternotomy was defined as the median sternotomy group. In order to balance the prognostic factors of the two groups, age and weight were used as matching factors for the propensity score method (caliper = 0.2, method = nearest). According to the demographic data from the right vertical axillary incision group, corresponding cases in the median sternotomy group was matched at the ratio of 1:2. Selected children were diagnosed as simple congenital atrial septal defect by preoperative transthoracic and transesophageal echocardiographyafter anesthesia. Preoperative infection index, cardiac function, liver and kidney function, coagulation function and other clinical parameters were normal. During the operation, the deair was monitored by transesophageal echocardiography to evaluate the correction of deformity. The same group of surgeons performed the operation for both groups. After the operation, the same group of clinicians completed the care and treatment. The effectiveness and safety outcomes in the right vertical axillary incision group were evaluated by comparing both operative and postoperative recovery and complications with those of the medial sternotomy group. Finally, the parents’ satisfaction with the postoperative appearance of their children was investigated through in-person and telephone follow-up. Clinical information of the participants is shown in Table 1.

**Table 1:** Clinical information of the study participants

	Right vertical axillary incision	Median sternotomy	U/Z/t/χ2	*P*
Gender (male/female)	9/9	20/16	0.386	0.7
Age (months)*	34.5 [14.0,59.8]	37.0[25.5,63.0]	283.5	0.46
Weight (kg)*	12.5[10.0,18.3]	12.3[10.0,18.0]	322	0.97
ASD diameter (mm)^#^	16.56 ± 1.05	18.58 ± 1.13	1.15	0.256
Hemoglobin (g/l)^#^	124.44 ± 2.27	122.47 ± 1.90	0.63	0.531
Preoperative NT-BNP (pg/ml)*	211.0[120.3, 403.0]	291.0 [120.8,582.9]	260	0.24
Incomplete right bundle branch block (Yes/No)	6/16	8/28	0.19	0.663
Arrhythmia (Yes/No)	2/16	1/35	1.59	0.208

*Data were presented as median [IQR]. ^#^ Data were presented as mean ± standard deviation. right vertical axillary incision group: surgery was performed with a small vertical incision through right armpit. Median sternotomy group: surgery was performed through median sternotomy. ASD: Atrial septal defect. NT-BNP: N-terminal pro-brain natriuretic peptide

### Right vertical axillary incision method

All the patients had a body weight within 6-30 kg, regardless of the type of atrial septal defect.

**Fig. 1:**
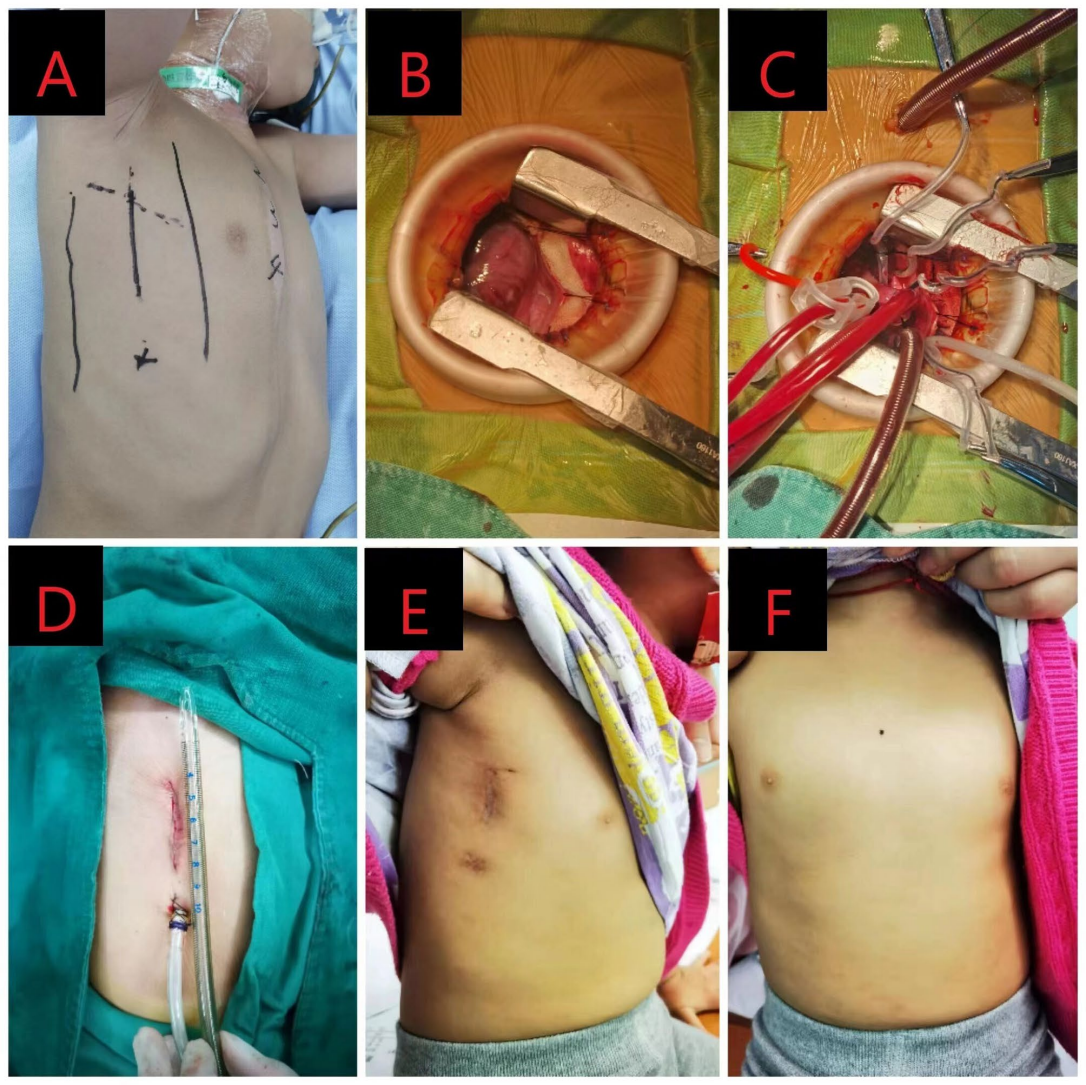
Right vertical axillary incision at different times A: right decubitus position before surgery; B: surgical field; C: cannulas inserted for cardiopulmonary bypass; D: length of axillary incision; E: axillary incision scar; F: front side of a patient after axillary incision heart repair

After endotracheal intubation under general anesthesia and transesophageal echocardiographyevaluation, the patient is placed in left lateral decubitus position. Theright chest israised by 70 degrees while theright arm is fixed over the head or hung on the head frame. A longitudinal incision in the mid-axillary line, about 4-5 cm, is made, and the chest is entered through the 4th intercostal space, pushing the right lung posteriorly. The pericardium is opened 1–2 cm in front of and parallel to the phrenic nerve.Pericardial stay stitches are made in the ascending aortic fold and the right pericardium to fully expose the heart. After heparinization, the right auricle is retracted caudally with a snare. The aortic root is retracted towards caudally with a right-angle clamp to expose the ascending aorta. the purse-strings are sutured, and a straight aortic cannula is inserted. A right-angle cannula is inserted directly into the superior vena cava to start cardiopulmonary bypass, and then a straight cannula is inserted into the inferior vena cava through an opening about 2–3 cm 6-7th intercostal space below the lower end of the skin incision. A right angle aortic cross clamp is applied, 20 ml/kg of cold crystalloid cardioplegia infused. The temperature is lowered to 31–34 degrees Celsius. The defect is repaired with continuous Gore-Tex patches with 6 − 0 polypropylene sutures through a right atrial incision. (Fig. 1) Routine mechanical assisted ventilation and anti-infection prophylaxis is provided postoperatively. (Fig. 1)

### Median sternotomy method

The patient is placed in the supine position, intubated under general anesthesia. After the evaluation of transesophageal echocardiography, a 6–8 cm is was made for sternotomy. After the sternum is opened with a spreader, the pericardium is cut in a T shape, suspended, and fixed on the chest wall to fully expose the heart. Under mild hypothermia cardiopulmonary bypass, a 6 − 0 polypropylenesuture and an autologous pericardium patch are used to repair the atrial defect. Routine mechanical assisted ventilation and anti-infection prophylaxis is provided postoperatively.

### Variables

The incision length, operative duration, cardiopulmonary bypass time, aortic cross clamp time, residual bubbles rate of right ventricular outflow tract/main pulmonary artery, postoperative ventilation duration, intensive care duration, drainage volume, indwelling duration of drainage tube, postoperative sedation and analgesia, postoperative hemoglobin, C-reactive protein (CRP), brain natriuretic peptide and incidences of chest wall deformitywere compared between the two groups. Postoperative cosmetic satisfaction was investigated during postoperative follow-ups.

### Statistical analysis

Shapiro-Wilk method is used to test the normality of the data. Normally distributed data were, described by means ± standard deviations and compared by t-test. Non-normally distributed data, were described by medians [interquartile range, IQR] and compared by nonparametric test. The classification data were compared by Chi-square test. SPSS 19.0 software(IBM Corp., Armonk, NY) was used for statistical analysis. P < 0.05 is considered statistically significant.

## Results

A total of 18 cases were included in the right vertical axillary incision group, 9 males and 9 females, with a median age of 34.5 [14.0, 59.8] months and a median weight of 12.5 [10.0, 18.3] kg. According to the propensity score method, 36 cases in median sternotomy group were matched from 124 children undergoing median sternotomy operation. This group includes 20 males and 16 females, with a median age of 37.0 [25.5, 63.0] months and a median weight of 12.3 [10.0, 18.0] kg. There were no significant differences in sex, age, and weight between the two matched groups. There were also no significant differences in the largest diameter of atrial septal defect, hemoglobin, N-terminal pro-brain natriuretic peptide (NT-BNP), right bundle branch block and arrhythmia in electrocardiography (ECG) results between the two groups before operation (Table 1).

**Fig. 2:**
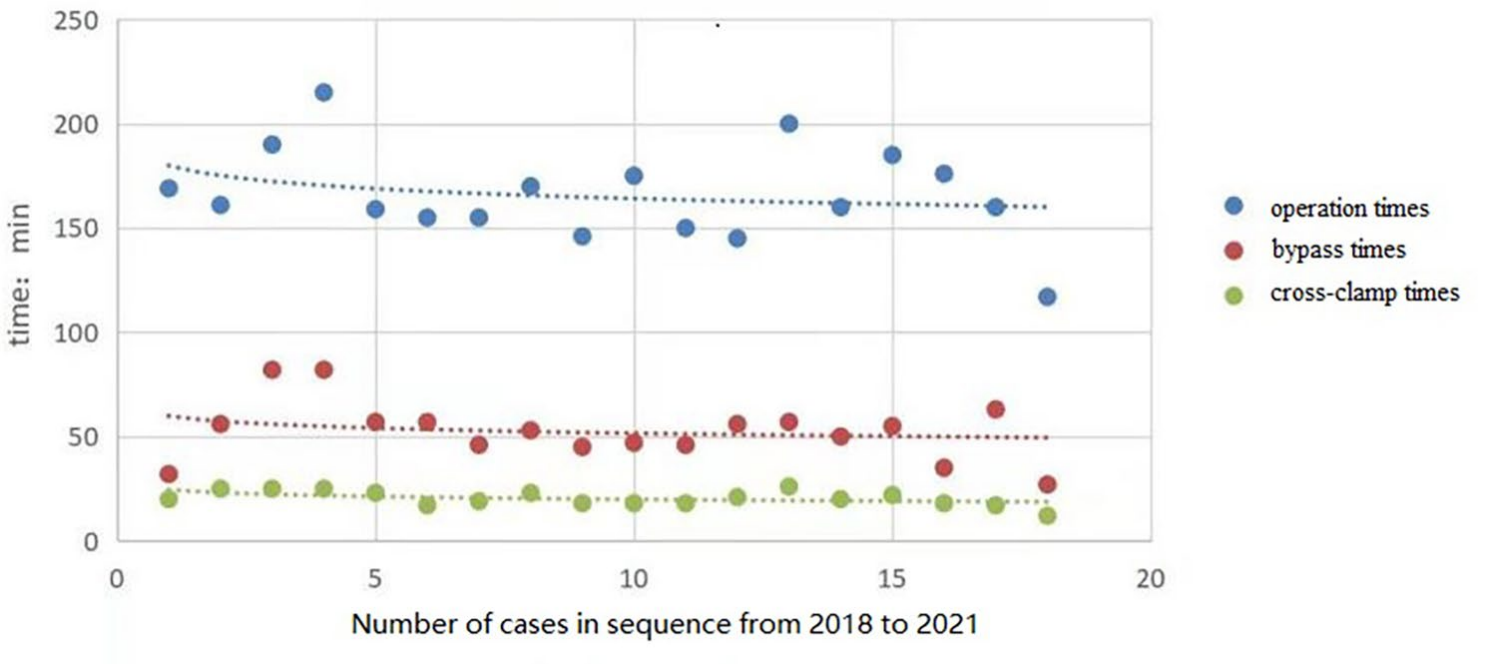
Operation, bypass and cross-clamp times of the 18 cases with axillary incision

With regard to the intraoperative conditions, the median bypass time of the right vertical axillary incision group was 54.0 [45.8, 57.0] minutes, which was longer than that of median sternotomy group (42.0 [39.0, 50.0] minutes, p = 0.007). The total operative duration of right vertical axillary incision group was 166.0 ± 22.5 min, which was longer than that of median sternotomy group (124.1 ± 21.7 min, p < 0.001). After routine deairing, the aortic cross-clamp was released and under transesophageal echocardiography assessment in both groups. While there were no cases with residual bubbles in the right vertical axillary incision group, the residual bubbles rate in the median sternotomy group was 44% (p = 0.001). There was no significant difference in aortic cross clamp time between the two groups (p = 0.311) (Table 2; Fig. 2).

**Table 2:** Operative parameters of the study participants

	Right vertical axillary incision group	Median sternotomy group	Z/t/χ2/χc2	*P*
Operation time (minutes)^#^	166 ± 22.5	124.1 ± 21.7	6.61	< 0.001
Bypass time (minutes)*	54.0[45.8,57.0]	42.0[39.0,50.0]	179.5	0.007
Cross-clamp time (minutes)*	20.0[18.0,23.0]	20.0[15.0,22.0]	269	0.311
Residual bubbles rate of right ventricular outflow tract (Yes/No)	0/18	16/20	11.37	0.001

*Data were presented as median [IQR]. ^#^ Data were presented as mean ± standard deviation

There were no significant differences in the durations of mechanical ventilation, intensive care, hospitalization, and indwelling duration of drainage tube. There were no significant differences in white blood cell count (WBC,) hemoglobin (Hb), CRP and NT-BNP on the first day after operation. There was also no significant difference between the two groups in the incidence of arrhythmia, right bundle branch block, pleural effusion and atelectasis indicated by bedside chest film after operation. The median incision length was 4.8 [4.0,5.0] cm in the right vertical axillary incision group, which was shorter than that of the medial sternotomy group (7.0 [7.0,8.0]cm, p < 0.001). The median drainage volume of the right vertical axillary incision group was 117.5 [92.8,152.8] ml, which was smaller than that of the median sternotomy group (151.5 [118.5,214.5] ml, p = 0.021). Both groups received analgesia pump routinely for postoperative analgesia. Two cases in the right vertical axillary incision group needed additional oral ibuprofen and/or chloral hydrate to assist sedation and analgesia, while 19 cases in the median sternotomy group required this procedure (p = 0.003). In the right vertical axillary incision group, the wound healed well one week after operation while there were 7 cases of incision infection or poor healing in median sternotomy group. The incidence of wound infection or poor healing after operation in the right vertical axillary incision group was less than that of the median sternotomy group (p = 0.047). No chest deformities, such as secondary pectus excavatum and carinatum after operation, were observed in the right vertical axillary incision group, while 9 cases in the median sternotomy group showed different degrees of local sternum bulge or depression. There was a significant difference in the incidence of postoperative thoracic deformity between the two groups (p = 0.029). Both groups were followed onemonth, three months, half a year, and then once a yearpostoperatively. Three months after operation, the patients or their families were interviewedwith the Vancouver Scar Rating Scale [18] upon clinic visit or via telephone. The results showed that parents in the right vertical axillary incision group were all satisfied with the postoperative cosmesis, while 9 cases in the median sternotomy group were dissatisfied (p = 0.018) (Table 3).

**Table 3:** Postoperative outcomes of the study participants

Postoperative data	Right vertical axillary incision group	Median sternotomy group	U/t/χ2/χc2	*P*
Postoperative reexamination results				
WBC (10^9/l)^#^	12.4 ± 2.4	13.3 ± 4.8	0.72	0.48
Hb (g/l)^#^	118.6 ± 10.9	114.0 ± 10.9	1.54	0.13
CRP (mg/dl)*	12.3[8.5,25.2]	13.9[9.8,21.0]	300	0.666
NT-BNP (pg/ml)*	1047.0[499.9,1472.0]	912.5[493.0,1683.0]	322	0.970
Arrhythmia (Yes/No)	1/17	2/34	0	0.71
Atrioventricular block (Yes/No)	4/14	4/32	1.174	0.244
Retention time of drainage tube (hours)^#^	88.6 ± 25.8	86.3 ± 31.3	0.27	0.791
Auxiliary ventilation time (minutes)*	127.5[120.0,155.0]	145.0[112.5,180.0]	295	0.599
Treatment time (hours)*	42.0[19.8,72.3]	41.8[21.3,45.0]	265.5	0.287
Postoperative drainage (ml)*	117.5[92.8,152.8]	151.5[118.5,214.5]	198.5	0.021
The time to pull out the drainage tube after operation was longer than 4 days (Yes/No)	5/13	10/36	0	0.62
Postoperative atelectasis (Yes/No)	1/17	6/30	1.31	0.25
Postoperative funnel chest (Yes/No)	0/18	1/35	0.51	0.667
Chicken breast after operation (Yes/No)	0/18	8/28	4.70	0.029
Postoperative sedation and analgesia (Yes/No)	2/16	19/17	8.77	0.00
Wound infection (Yes/No)	0/17	7/29	4.0	0.047
Length of stay (days)*	11.0[9.8,13.3]	9.5[8.0,13.0]	248.5	0.165
Operative incision length (cm)*	4.8[4.0,5.0]	7.0[7.0,8.0]	0	< 0.001
Postoperative appearance (Satisfactory/Unsatisfactory)	18/0	27/9	5.4	0.018^*^

*Data were presented as median [IQR]. ^#^ Data were presented as mean ± standard deviation. WBC: white blood cells; Hb: hemoglobin; CRP:C-reactive protein; BNP: N-terminal pro-brain natriuretic peptide

## Discussion

In the treatment of congenital heart disease, the primary task of surgical intervention is to complete an optimal repair to ensure the longest life expectancy and the best quality of life [2, 19]. While early surgical treatment of congenital atrial septal defect has achieved satisfactory results, the psychological burden brought by conventional median sternotomy scar to children, teenagers and adult women cannot be ignored. In recent years, with the continuous improvement of related technologies of cardiac surgery, minimally invasiveness and postoperative aesthetics have become the research focus. More cardiac surgeons are exploring ways to minimize surgical trauma and improve postoperative aesthetics [1, 6, 8, 9]. The success rate and long-term results of open-heart surgery through a right axillary incision is more satisfactory than those obtained through median sternotomy. This finding is consistent with a previous report of Hannah et al. [20]. Additionally, patients receiving surgeries via right axillary incision have a higher quality of life after operation [17], which has been widely used in the clinics as a minimally invasive operation [8, 16, 21−26]. 

Since 2018, we have completed the reparation of simple atrial septal defects through the right vertical axillary incision. Compared with the traditional median sternotomy surgery, there are some limitations in the choice of age and weight. The surgical view would be limited in small children. In contrast, if the age and weight are too large, the surgical field is very deep, making operation technique challenging. Due to medical insurance policy at our institution, one lung ventilation would increase the cost of hospitalization. For the patient in this group, the exposure in axillary incision group was acceptable without one lung ventilation, During the same period, we also carried out the surgical treatment of other conditions such as ventricular septal defect, partial anomalous pulmonary venous drainage, etc. and have also achieved good results.

The selection of surgical instruments, cardiopulmonary bypass tubings, and intercostal space also needexperience. In this study, there were no special requirements for the size and type of atrial septal defects in the right vertical axillary incision group. Their related routine blood test results, blood biochemistry and ECG data before operation are similar to those of the medial sternotomy group.

The lack of significant difference in aortic cross-clamp time between the two groups shows that different surgical incision approaches do not increase the difficulty of defect repair. The cardiopulmonary bypass time and total operative duration of the right vertical axillary incision group were longer than those of the median sternotomy group, indicating that the exposure of surgical field, surgical skills, and cannulationthrough the right vertical axillary incisionare more difficult. Since the learning curve during this study has not reached plateau, the operative durationwas not optimal. Our preliminary experience in this study, however, has suggested a continuous reduction in the operative time andcross-clamping time as experience accumulated. A comparative study of several different incisions by Praveen Reddy Bayya [27] shows that these problems could be overcome by skilled surgeons.

The right vertical axillary incision is minimally invasive, which does not require special technology and equipment. Compared to the traditional median sternotomy, the right vertical axillary incision has a reduced incision length and a hidden scar. The incidence of wound infection or poor healing and the subcutaneous tissue in the right vertical axillary incision group were significantly less than that in the median sternotomy group. After operation, it is easy to affect wound healing by drooling, drinking water, drinking milk or eating, or scratching if it is sternotomy. However, the right vertical axillary incision is hidden, not easy to be contaminated. After a sternotomy, it is easy to have a localized bulge or depression of sternum in different degrees. In contrast, in children with the right vertical axillary incision, thoracic bony structure is not impaired, resulting less wound pain or thoracic deformity duringhealing. Therefore, this approach is superiorfor children’s long-term mental health [17].

In this study, the left and right hearts of children with right vertical axillary incision were deaired thoroughly after routine deair during the operation. No residual bubbles were found in the left and right hearts of children with right vertical axillary incisionby intraoperative transesophageal echocardiography, while the residual bubble rate of children with medial sternotomy incision was higher. This indicates that the posture of the right vertical axillary incision group was better fordeairing during the operation, hence reduction or complete elimination of air embolism. The drainage volume in children with right vertical axillary incision after operation is significantly less than that of children with medial sternotomy incision, indicating that children in the former group had smaller wound area, betterhemostasis upon chest closure, and lower incidence of active bleeding. Although the times of cardiopulmonary bypass and operation in children with right vertical axillary incisionwere longer than those of children with median sternotomy incision, there were no significant differences in mechanical ventilation duration,intensive care duration. The surgical and long-term results of the right vertical axillary incisionweremore favorable than those of median sternotomy group. The scars were cosmetically satisfactory at the 6-month postoperative follow-up, and no complications related to thoracic deformity or movement impairment of the right upper extremity were identified. These findings are consistent with a study by Hannah et al. [19]. More importantly, patients’ satisfaction with the treatment and cosmesis was 100% after the operation [10, 11]. The purpose of this approach is to reduce surgical trauma, to accelerate postoperative rehabilitation, and to provide better cosmesis [3–5, 17, 28, 29] for children with CHD to the greatest extent without compromising the surgical results. Therefore, this approach is not only feasible and safe for open-heart surgery in children with CHD but also easily accepted by the families.

During the same study period, we also used lower partial sternotomy. Because the sternum is only partially incised, the lower sternum tends to protrude outward during healing process, and the scar is still visible in the front chest [17]. Anterolateral thoracotomy for open heart surgery, which has to mobilize large muscle area and soft tissue, may lead to muscle deformation, nipple sensitivity reduction, breast and pectoralis muscle dysplasia and cosmetic problems [16, 22, 23]. The technical requirements of complete VATS-assisted and robot-assisted open-heart surgery are demanding, and the operative space of infants is limited, so it is difficult to implement in small children. Once an accident occurs during the operation, it may be necessary to switch to open chest surgery, and the cost is too high for most families to afford [17, 20]. There is no need to prepare special technology and equipment, and the expenses for patients are not increased.

Our study may have some potential limitations. As an observational retrospective and a single-institution study, our sample size is small. In order to minimize the influence of selection bias and other confounding factors on the results, we conducted a case-matching analysis. While the results of this study showed that the right vertical axillary incision approach was a good alternative to the median sternotomy approach, the operative duration of the former needs to be improved with additional clinical experience. Also, the follow-up period is relatively short. Longer term follow-up is warrant for further evaluation of this surgical approach.

## Conclusion

While the right vertical axillary incision surgery is technically challenging, it can effectively reduce surgical trauma, accelerate the postoperative rehabilitation and provide better cosmetic results. In simple congenital heart diseases such as atrial septal defect, this approach is a promising alternative to the traditional median sternotomy incision surgery.

## Data Availability

Data can be provided upon request.

## List of abbreviations

ASD: atrial septum defect
CHD: congenital heart disease
CRP: C-reactive protein
ECG: electrocardiography
Hb: hemoglobin
IQR: interquartile range
NT-BNP: N-terminal pro-brain natriuretic peptide
WBC: white blood cells
